# Supplementary material for: Application of computer vision in assessing crop abiotic stress: A systematic review
Source: PLoS One. 2023 Aug 23;18(8):e0290383. doi: 10.1371/journal.pone.0290383 (PMC10446212; doi:10.1371/journal.pone.0290383)
Supplement: S2 File — This file contains the whole search string for each database considered, as well as the extra criteria used to retrieve relevant entries. (DOCX) [file pone.0290383.s002.docx]

| **Database** | **Search String** | **Additional Tags** |
| --- | --- | --- |
| Scopus | TITLE-ABS-KEY ( ( "crop" OR "plant" OR "soil organic matter" OR "pH" OR "water" ) AND ( "nutri*" OR "npk" OR "element*" OR "abiotic stress" ) AND ( "defici*" OR "short*" OR "inadeq*" OR "insuffi*" ) AND ( "estima*" OR "predict*" OR "recogn*" OR "detect*" OR "assess*" OR "analysis" ) AND ( ( ( "machine" OR "deep" ) AND "learning" ) OR "artificial intelligence" OR "image process*" ) ) AND PUBYEAR > 2011 AND ( LIMIT-TO ( SRCTYPE , "j" ) ) AND ( LIMIT-TO ( PUBSTAGE , "final" ) ) AND ( EXCLUDE ( PUBYEAR , 2023 ) ) AND ( LIMIT-TO ( LANGUAGE , "English" ) ) AND ( LIMIT-TO ( DOCTYPE , "ar" ) ) | N/A |
| Web of Science | TS=(( "crop" OR "plant" OR "soil organic matter" OR "pH" OR "water" ) AND ( "nutri*" OR "npk" OR "element*" OR "abiotic stress" ) AND ( "defici*" OR "short*" OR "inadeq*" OR "insuffi*" ) AND ( "estima*" OR "predict*" OR "recogn*" OR "detect*" OR "assess*" OR "analysis" ) AND ( ( ( "machine" OR "deep" ) AND "learning" ) OR "artificial intelligence" OR "image process*" )) | Publication Years:  2012-2022 Document Type: Article Language: English |
| ScienceDirect | ( "crop") AND ( "nutrient" OR "npk" OR "abiotic stress" ) AND ( "machine learning" OR "deep learning" OR "artificial intelligence") | Year: 2012-2022 Article Type: Research articles Subject area: Agricultural and Biological Sciences |
| BASE | (crop nutritional deficiency) AND (machine learning OR deep learning OR artificial intelligence) doctype:(121 15 19) year:[2012 TO 2022] | Language: English Subject: Agriculture |
| Wiley Online Library | ( "crop" OR "plant" OR "soil organic matter" OR "pH" OR "water" ) AND ( "nutri*" OR "npk" OR "element*" OR "abiotic stress" ) AND ( "defici*" OR "short*" OR "inadeq*" OR "insuffi*" ) AND ( "estima*" OR "predict*" OR "recogn*" OR "detect*" OR "assess*" OR "analysis" ) AND ( ( ( "machine" OR "deep" ) AND "learning" ) OR "artificial intelligence" OR "image process*" ) | Publication Date:  2012-2022 Publication Type: Journals Subject: Agriculture |

**Table: Exact search strings across selected engines with additional tags**
