# Supplementary material for: Application of computer vision in assessing crop abiotic stress: A systematic review
Source: PLoS One. 2023 Aug 23;18(8):e0290383. doi: 10.1371/journal.pone.0290383 (PMC10446212; doi:10.1371/journal.pone.0290383)
Supplement: S4 File — This file includes the specific reasoning for each study that was excluded after accessing the complete texts. (DOCX) [file pone.0290383.s004.docx]

**Table: Criteria of the omitted studies and their justifications**

| **Author (Year)** | **Title** | **Exclusion Criteria** | **Details** |
| --- | --- | --- | --- |
| Asaari et al. (2019) | Analysis of Hyperspectral Images for Detection of Drought Stress and Recovery in Maize Plants in A High-Throughput Phenotyping Platform | Did not use deep learning | Deep learning wasn't part of the research work. Moreover, the authors did not utilize photos as input, but rather water stress spectrum data. |
| Navarro et al. (2022) | Sorting Biotic and Abiotic Stresses on Wild Rocket by Leaf-Image Hyperspectral Data Mining with An Artificial Intelligence Model | Did not use deep learning | While an Artificial Neural Network was employed in the study, only one layer was considered, hence it cannot be considered as deep learning. Moreover, the authors did not utilize photos as input, but rather spectrum data derived from hyperspectral images. |
| Romualdo et al. (2014) | Use of Artificial Vision Techniques for Diagnostic of Nitrogen Nutritional Status in Maize Plants | Did not use deep learning | The study extracted characteristics using volumetric fractal dimension and Gabor wavelets, however no algorithms for deep learning were applied. |
| Haider et al. (2021) | A Computer-Vision-Based Approach for Nitrogen Content Estimation in Plant Leaves | Did not use deep learning | The work presented a new image processing-based approach for quantifying the nitrogen content of a leaf photograph; however, no deep learning algorithm was being used. |
| Guerrero et al. (2018) | Development of a Computational Tool to Identify Nutritional Deficiencies in Plants (Passiflora tripartita var. mollissima) | Did not use deep learning | The researchers created a MATLAB application to segment and categorize crop photos of nutritional deficiencies; however, no deep learning algorithms or artificial intelligence were involved. |
| Aleksandrov et al. (2022) | Identification of Nutrient Deficiency in Plants by Artificial Intelligence | Did not use images as input | The study used prompt fluorescence and associated transient curve information as inputs, rather than photographs. Also, deep learning wasn't considered. |
| Atefi et al. (2019) | In Vivo Human-Like Robotic Phenotyping of Leaf Traits in Maize and Sorghum in Greenhouse | Did not use images as input | The research utilized temperature and leaf visible and near infrared spectra as inputs, but not visuals. |
| Feng et al. (2022) | Multitask Learning of Alfalfa Nutritive Value from UAV-Based Hyperspectral Images | Did not use images as input | The article made use of time-series data. Deep learning wasn't taken into account either. |
| Abdalla et al. (2021) | Nutrient Status Diagnosis of Infield Oilseed Rape via Deep Learning-enabled Dynamic Model | Did not use images as input | Instead of only employing photos as input, this study also used time-series data. |
| Chore et al. (2022) | Nutrient Defect Detection in Plant Leaf Imaging Analysis Using Incremental Learning Approach with Multifrequency Visible Light Approach | Did not use images as input | Despite using a deep learning architecture, the authors provided the system spatial and temporal light parameters as well as visuals. |
| Chang et al. (2021) | Using a Hybrid Neural Network Model DCNN-LSTM for Image-Based Nitrogen Nutrition Diagnosis in Muskmelon | Did not use images as input | Although the researchers employed deep learning (a DCNN-LSTM hybrid model), they utilized thermal effectiveness and photosynthetically active radiation (TEP) data in addition to photographs as input of the system. |
| Angin et al. (2020) | AgriLoRa: A Digital Twin Framework for Smart Agriculture | Not related to abiotic stress detection | The research mentioned recognizing crop nutrient deficiencies in the abstract, but it solely dealt with diseases present in PlantVillage dataset. |
| ElManawy et al. (2022) | HSI-PP: A Flexible Open-Source Software for Hyperspectral Imaging-Based Plant Phenotyping | Not related to abiotic stress detection | HSI-PP is a hyperspectral image processing tool for plant phenotyping. However, the study did not specifically address classification or detection of abiotic stresses in crops. |
| Wiegman et al. (2022) | Intra-Canopy Sensing Using Multi-Rotor sUAS: A New Approach for Crop Stress Detection and Diagnosis | Not related to abiotic stress detection | This paper concentrates on the Stinger platform's proof-of-concept, which consists of miniature unmanned aerial vehicles, and its capacity to give data for deep learning algorithms that may recognize nutritional deficiencies or other abiotic stresses. But, at this point, the study is primarily concerned with the theoretical functioning of the system. |
| Contreras-Medina et al. (2012) | Smart Sensor for Real-Time Quantification of Common Symptoms Present in Unhealthy Plants | Not related to abiotic stress detection | Instead of focusing just on nutrient shortages, the study introduced new image processing-based algorithms that can recognize symptoms which might potentially be attributed to pathogens. Furthermore, the algorithms developed have little to do with deep learning or artificial intelligence in general. |
| Zhu et al. (2022) | Improving rice nitrogen stress diagnosis by denoising strips in hyperspectral images via deep learning | Not related to abiotic stress detection | Rather than abiotic stress classification or recognition, the research concentrates on the reduction of strip noise, i.e., de-striping of hyperspectral images. |
| Lewis et al. (2020) | Classification and Detection of Nutritional Deficiencies in Coffee Plants Using Image Processing and Convolutional Neural Network (CNN) | Potential predatory or questionable publisher/journal | With effect from 2020, Scopus stopped including the journal. Furthermore, neither the journal nor the publisher is included in DOAJ, COPE, or OASPA. |
| Adesanya et al. (2022) | Classification of Nitrogen Deficiency for Maize Plants Using Deep Learning Algorithms on Low-End Android Smartphones | Potential predatory or questionable publisher/journal | The journal's website does not offer information about where it is indexed. It charges article processing fees even before the review process begins. There are also spelling mistakes on the webpage and the journal/publisher is not listed in COPE or OASPA. |
| Marcelo et al. (2020) | Corzea: Portable Maize (Zea Mays L.) Nutrient Deficiency Identifier | Potential predatory or questionable publisher/journal | With effect from 2020, Scopus stopped including the journal. Furthermore, neither the journal nor the publisher is included in DOAJ, COPE, or OASPA. |
| Sathyavani et al. (2021) | Detection of Plant Leaf Nutrients Using Convolutional Neural Network Based Internet of Things Data Acquisition | Potential predatory or questionable publisher/journal | With effect from 2022, Scopus stopped including the journal. Furthermore, neither the journal nor the publisher is included in DOAJ, COPE, or OASPA. |
| Lavanya et al. (2022) | Deep Learning for Identification of Plantnutrient Deficiencies | Potential predatory or questionable publisher/journal | Although the journal is focused on pharmaceuticals, it includes works that are entirely beyond of its scope. Furthermore, neither the journal nor the publisher is included in DOAJ, COPE, or OASPA. |
| Leena et al. (2018) | Classification of Macronutrient Deficiencies in Maize Plant Using Machine Learning | Did not exceed the critical appraisal of methodological quality threshold | Three reviewers (NAO, MNU, MLB) rated the study a methodological quality score of less than or equal to 2. |
| Shidnal et al. (2021) | Crop Yield Prediction: Two-Tiered Machine Learning Model Approach | Did not exceed the critical appraisal of methodological quality threshold | Three reviewers (NAO, MNU, MLB) rated the study a methodological quality score of less than or equal to 2. |
| Dal Prá et al (2020) | Nutritional Evaluation of Brachiaria Brizantha cv. Marandu Using Convolutional Neural Networks | Did not exceed the critical appraisal of methodological quality threshold | Three reviewers (NAO, MNU, MLB) rated the study a methodological quality score of less than or equal to 2. |
| Wulandhari et al. (2019) | Plant Nutrient Deficiency Detection Using Deep Convolutional Neural Network | Did not exceed the critical appraisal of methodological quality threshold | Three reviewers (NAO, MNU, MLB) rated the study a methodological quality score of less than or equal to 2. |
| Kusanur et al. (2021) | Using Transfer Learning for Nutrient Deficiency Prediction and Classification in Tomato Plant | Did not exceed the critical appraisal of methodological quality threshold | Three reviewers (NAO, MNU, MLB) rated the study a methodological quality score of less than or equal to 2. |
| Zhou et al. (2021) | An Automatic Non-Invasive Classification for Plant Phenotyping by MRI Images: An Application for Quality Control on Cauliflower at Primary Meristem Stage | Did not exceed the critical appraisal of methodological quality threshold | Three reviewers (NAO, MNU, MLB) rated the study a methodological quality score of less than or equal to 2. |
| Waheed et al. (2022) | Deep Learning Based Disease, Pest Pattern and Nutritional Deficiency Detection System for “Zingiberaceae” Crop | Did not exceed the critical appraisal of methodological quality threshold | Three reviewers (NAO, MNU, MLB) rated the study a methodological quality score of less than or equal to 2. |
