# Supplementary figures and images for: Application of computer vision in assessing crop abiotic stress: A systematic review
Source: PLoS One. 2023 Aug 23;18(8):e0290383. doi: 10.1371/journal.pone.0290383 (PMC10446212; doi:10.1371/journal.pone.0290383)

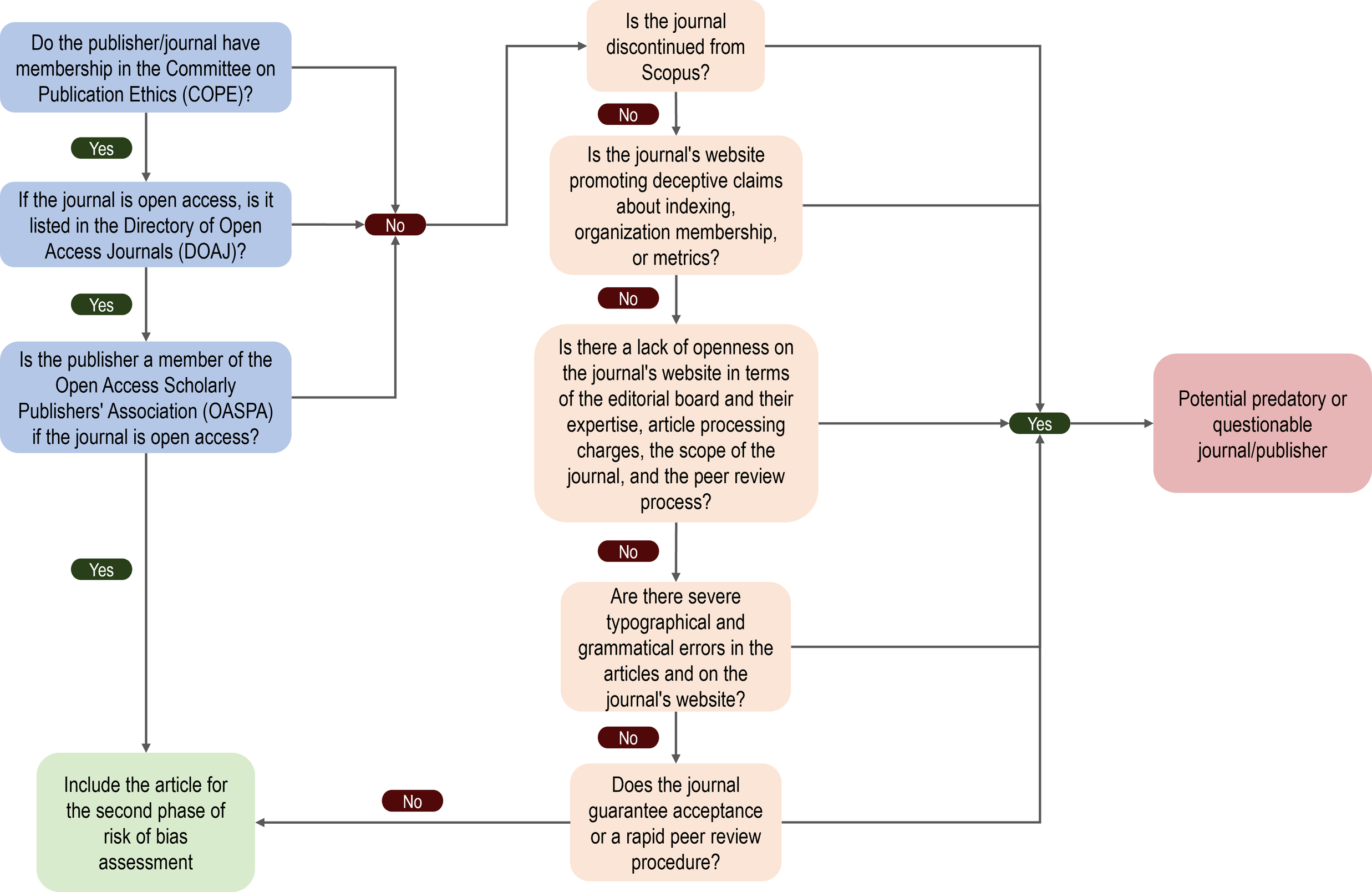

Supplement: S1 Fig — Using this chart, we evaluated the publishers or journals depending on their expulsion from Scopus coverage for editorial malpractices, false and misleading information regarding indexing and memberships on their websites, a lack of overall transparency, and a variety of other factors. (TIF) [file pone.0290383.s007.tif]
