# Supplementary material for: Application of computer vision in assessing crop abiotic stress: A systematic review
Source: PLoS One. 2023 Aug 23;18(8):e0290383. doi: 10.1371/journal.pone.0290383 (PMC10446212; doi:10.1371/journal.pone.0290383)
Supplement: S1 Table — Using this table, we assessed the findings of the reviewed studies, namely the inter-class precision and recall, while taking into account GRADE’s five identified categories: risk of bias, imprecision, inconsistency, indirectness, and publication bias. (DOCX) [file pone.0290383.s008.docx]

| **Quality level** | **Definition** |
| --- | --- |
| High | We are very confident that the true effect lies close to that of the estimate of the effect. |
| Moderate | We are moderately confident in the effect estimate: The true effect is likely to be close to the estimate of the effect, but there is a possibility that it is substantially different. |
| Low | Our confidence in the effect estimate is limited: The true effect may be substantially different from the estimate of the effect. |
| Very Low | We have very little confidence in the effect estimate: The true effect is likely to be substantially different from the estimate of the effect. |

**Table: The four levels of evidence used in the GRADE profile**
