# Supplementary material for: Application of computer vision in assessing crop abiotic stress: A systematic review
Source: PLoS One. 2023 Aug 23;18(8):e0290383. doi: 10.1371/journal.pone.0290383 (PMC10446212; doi:10.1371/journal.pone.0290383)
Supplement: Attachment — Submitted filename: Response to Reviewers.pdf [file pone.0290383.s009.pdf]

# Response to Reviewers

***Reviewer #1:*** *The review article entitled Application of computer vision in assessing crop abiotic stress: A systematic review by Nabil Anan Orka et al is very well written. The title of the manuscript is quite good and can attract readers attention. I would recommend acceptance of the manuscript.*

We would like to convey our sincerest gratitude for the kind words. It will be a privilege to represent a prestigious journal such as PLOS ONE.

***Reviewer #2:*** *Following an extensive screening and analysis of papers published in the past decade, a comprehensive total of 2399 records from five search sources underwent screening for this study. Ultimately, a final selection of 14 publications was yielded. The total publication records offer valuable insights into 11 abiotic stressors that impact seven distinct crops. It is worth noting that a notable majority of the selected publications (8 out of 14) originate from 2022, with no publications spanning the years 2012 to 2017. There are some contents that are not very clear to me, please clarify them in writings or figures.*

*1) In Figure 1, there are certain factors that seem unclear, such as "Report not retrieved (n=2)" and "Did not use deep learning (n=5)". Could you please provide a more detailed explanation regarding why these publications were excluded from the list after the screening process?*

We adhered to the PRISMA 2020 guidelines to conduct the systematic review. We chose the research articles for the systematic review (14 in total) by meticulously sticking to the rules and outlining the selection approach in detail. It should be emphasized that we did not omit studies from 2012 to 2017 on purpose, but rather that the deep learning boom in the stress identification space began late. When reviewers are unable to access articles from their individual online addresses, the issue must be indicated in the selection method/workflow figure, according to the standards. As a result, we indicated this using "Reports not retrieved," followed by the number of articles that could not be acquired, using the structure recommended by the PRISMA rules. In addition, we mentioned the number of inaccessible articles on lines 177-178. Our systematic review is exclusively geared towards image-based deep learning frameworks for crop abiotic stress assessment. As such, on lines 24-37, we explained why we chose deep learning, focusing on its advantages over traditional machine learning methods. Deep learning can recognize complicated patterns and extract significant information through minimal engineering, and in certain cases, without the need for human supervision. Moreover, we pointed out that the use of deep learning in the eligibility criteria section, namely the inclusion criterion on lines 54-56. Hence, we excluded papers that did not utilize deep learning. Furthermore, we provided a supporting information file called S4 that covers the reasoning behind each manuscript that was rejected during the second screening step.

*2) Regarding Figure 2, 3, and 4, it appears that only the final yield of 14 publications was used to generate Figures 2 and 3. Are the 2399 total records distributed similarly across different years and plant species? Conversely, it seems that the total records were used to create Figure 4, aiming to identify the crop abiotic stressors. Therefore, I suggest using the total records to create Figures 2 and 3 as well. Similarly, the yield of the 14 selected publications could be used to create Figure 4.*

First and foremost, please accept our sincere apologizes for any misunderstandings or ambiguity. Figures 2, 3, and 4 illustrate the yield of the 14 papers considered for the systematic review. Unfortunately, our phrasing accentuated the vagueness. As a result, we modified the caption of figure 4. We evaluated 2399 papers in total, resulting in 14 publications for the final systematic review. All of the figures and tables in the results section are founded on the selected 14 articles, not the entirety of 2399 articles. The guidelines of PRISMA's screening system are composed in such a way that we cannot depict the distribution of papers on crops and abiotic stresses without reading the manuscripts. To avoid biases, accessing the main content of the article is prohibited at the first round of screening. We would thus be violating the protocol if we presented the entire set of 2399 articles in the results section. Again, we apologize for any confusion.

*3) Grammar error: “Several research” row 249, can be change to “several research studies”*

We sincerely apologize for overlooking the grammatical problem in our initial submission. We rectified it right away. Thank you for your valuable suggestion.

*4) Some references are in different styles. For example: reference 7 is "nature" while reference 16 is "Nature". Please correct this type of error accordingly.*

We apologize for overlooking the referencing problems. Thank you very much for bringing these items to our attention. Since then, we have followed the requirements on the PLOS ONE website and corrected them. We also changed the names of the journals to match those listed in the National Center for Biotechnology Information (NCBI) databases.
